# CXCL10 rs8878 identifies a genotype-associated immune phenotype linked to T-lymphocyte preservation and survival in sepsis

**DOI:** 10.3389/fimmu.2026.1887361

**Published:** 2026-07-17

**Authors:** Birte Dyck, Andrea Witowski, Thilo Bracht, Malte Bayer, Patrick Thon, Dominik Ziehe, Tim Rahmel, Matthias Unterberg, Britta Westhus, Lars Palmowski, Hartmuth Nowak, Stefan Felix Ehrentraut, Jennifer Orlowski, Alexander von Busch, Alexander Zarbock, Nina Babel, Moritz Anft, Dietrich Henzler, Michael Adamzik, Lars Bergmann, Barbara Sitek, Björn Koos, Katharina Rump

**Affiliations:** 1 Department of Anesthesiology, Intensive Care Medicine and Pain Therapy, Center of perioperative precision medicine, Ruhr University Bochum, Knappschaft Kliniken University Hospital Bochum, Bochum, Germany; 2 Ruhr University Bochum, Knappschaft Kliniken University Hospital Bochum, Department of Anesthesiology, Intensive Care Medicine and Pain Therapy, Bochum, Germany; 3 Department of Anesthesiology, Intensive Care Medicine and Pain Therapy, Center for Proteomics and Metabolomics, Ruhr University Bochum, Knappschaft Kliniken University Hospital Bochum, Bochum, Germany; 4 Department of Anesthesiology, Intensive Care Medicine and Pain Therapy, Center for Artificial Intelligence, Medical informatics and Data Science, Ruhr University Bochum, Knappschaft Kliniken University Hospital Bochum, Bochum, Germany; 5 Department of Anesthesiology and Intensive Care Medicine, University Hospital Bonn, Bonn, Germany; 6 Department of Anesthesiology, Intensive Care and Pain Medicine, University of Münster, Münster, Germany; 7 Ruhr Universität Bochum, Medizinische Klinik I, Universitätsklinik Marien Hospital Herne, Herne, Germany; 8 Klinikum Herford, Department of Anesthesiology, Surgical Intensive Care, Emergency and Pain Medicine, Ruhr University Bochum, Herford, Germany

**Keywords:** CD8^+^ lymphocytes, CXCL10 (C-X-C motif ligand 10), phenotypes, rs8878, sepsis, SepsisDataNet.NRW, survival, T cell

## Abstract

**Background:**

Sepsis is characterized by a dysregulated host response to infection, leading to concurrent hyperinflammation and immunosuppression, including profound alterations in T lymphocyte homeostasis. The chemokine CXCL10, an interferon-γ-inducible mediator of T cell trafficking, has been implicated in immune activation and tissue injury. However, it remains unclear whether genetic variation in CXCL10 contributes to T cell dysregulation and clinical outcomes in sepsis.

**Methods:**

In a prospective cohort of septic patients (n=278), we analyzed CXCL10 rs8878 genotypes, circulating immune cell counts, cytokine concentrations, and CXCL10 protein and mRNA expression in whole blood. Associations between genotype, immune parameters, plasma proteomics and 30-day survival were assessed using group comparisons and Kaplan-Meier analyses. Correlation analyses were performed to evaluate relationships between CXCL10 concentrations, cytokines, and clinical parameters.

**Results:**

Variants in the CXCL10 gene were associated with T cell dysregulation. Carriers of the rs8878 AA genotype exhibited higher circulating T cell counts and improved survival compared with G-allele carriers. Higher total and CD8^+^ T cell counts were significantly associated with improved survival. Among non-survivors, AA-genotype carriers showed increased CXCL10 mRNA expression, indicating genotype-dependent regulation of CXCL10 expression under conditions of fatal disease progression. CXCL10 concentrations on day 1 were positively correlated with multiple inflammatory cytokines, including IL-6, IL-8, IL-10, IL-18, MCP-1, IFN-γ, and interferon-α2, and inversely correlated with total T cell counts, supporting a link between CXCL10, systemic inflammation, and T cell depletion. No significant associations were observed between CXCL10 genotype and plasma proteomics and routine clinical parameters.

**Conclusion:**

The CXCL10 rs8878 genotype is associated with T cell dynamics and 30-day survival in sepsis, suggesting a genotype-dependent modulation of the adaptive immune response. While the AA genotype is linked to preserved T cell counts and improved outcomes, increased CXCL10 expression in non-survivors points to a context-dependent role in inflammation-driven immune dysregulation. These findings identify CXCL10 as a potential biomarker for risk stratification and a candidate target for immunomodulatory therapies in sepsis.

## 1 Introduction

Sepsis is a life-threatening syndrome resulting from a dysregulated host response to infection and remains a major cause of morbidity and mortality in critically ill patients worldwide (1). During sepsis, profound alterations occur in both the innate and adaptive immune system (2). A hallmark of sepsis is immune dysfunction, including profound alterations in adaptive immunity (3). Among immune cells, T lymphocytes (T cells) play a central role in orchestrating pathogen clearance and maintaining immune homeostasis (3). Reduced circulating T cell counts during sepsis have been consistently associated with impaired immune competence and worse clinical outcomes (4).

Alterations in T cell numbers and function during sepsis are closely linked to dysregulated chemokine signaling, particularly involving CXCL10 (C-X-C motif chemokine ligand 10; also known as interferon-γ-induced protein 10, IP-10). Chemokines, together with acute-phase reactants, are among the key molecular mediators associated with sepsis. In septic patients, several chemokines of the CC motif family measured in serum or plasma samples(including CCL1, CCL2, CCL8, and CCL20) as well as CXC motif chemokines (such as CXCL8, CXCL10, and CXCL12), along with various cytokines, are significantly increased compared to healthy controls (5).

CXCL10 is a chemokine induced by interferon-γ (IFN-γ) and other pro-inflammatory cytokines and mediates T cell trafficking to sites of inflammation (6). It binds to the receptor CXCR3, which is highly expressed on activated CD8^+^ cytotoxic T cells, Th1 CD4^+^ T cells, and NK cells (6). Elevated CXCL10 levels have been observed in septic patients, and its expression has been linked to both immune activation and organ injury (7). They have therefore been associated with disease severity, progression to septic shock, and poor outcome, although no consistent cut-off value has been established and concentrations vary depending on timing and methodology (8). As mentioned above, sepsis is also characterized by profound T cell lymphopenia, and while a direct clinical correlation between CXCL10 levels and absolute T cell counts has not been consistently demonstrated, experimental and immunological evidence suggests that high CXCL10 levels may promote CXCR3-dependent T cell redistribution, exhaustion, and apoptosis, thereby contributing to sepsis-associated immunosuppression (8).

Variations in CXCL10 expression and function have been implicated in several infectious, inflammatory, and malignant diseases (9, 10). Investigating single nucleotide polymorphisms (SNPs) in the CXCL10 gene offers insight into inter-individual differences in immune response, disease susceptibility, progression, and outcome (11).

Several studies show associations between CXCL10 promoter or untranslated region polymorphisms and disease. Among these variants, the 3′ untranslated region SNP rs8878 has been associated with altered CXCL10 expression and clinical outcomes in inflammatory diseases, including rheumatoid arthritis (12). Therefore, rs8878 represents a biologically plausible candidate variant for investigating the impact of CXCL10 genetics on immune dysregulation and outcome in sepsis. However, the impact of CXCL10 genetic variation on T cell abundance and survival in sepsis has not been fully elucidated.

Despite extensive research on the role of T cells and CXCL10 in sepsis, there is limited evidence connecting CXCL10 genotype, adaptive immune response, and clinical outcome. Understanding this relationship could provide mechanistic insights into the immune regulation in sepsis and identify potential prognostic biomarkers.

Therefore, in this study, we aimed to investigate the association between CXCL10 rs8878 genotype, circulating T lymphocyte counts, and 30-day survival outcomes in septic patients. Additionally, we assessed CXCL10 mRNA expression in non-survivors to explore potential genotype-dependent mechanisms underlying immune response and mortality. We hypothesized that the CXCL10 rs8878 polymorphism contributes to inter-individual differences in the immune response during sepsis and that AA-genotype carriers would exhibit enhanced preservation of circulating T-cell populations, altered CXCL10 expression, and improved 30-day survival compared with AG/GG genotype carriers.

## 2 Materials and methods

### 2.1 Study design and conceptual overview

In total, 278 patients from the prospective, multicenter SepsisDataNet.NRW cohort (185 patients from clinic A, 32 patients from clinic B, 18 patients from clinic C, 22 patients from clinic D, 14 patients from clinic E, 4 patients from clinic F and 3 patients from clinic G) were included. Clinical data, blood samples, and follow-up information were collected prospectively according to the study protocol. The analyses presented in the current manuscript, including genotyping, flow cytometry, and gene expression analyses, were subsequently performed using the collected biospecimens and data. Patients were recruited consecutively between March 1, 2018, and May 31, 2022. Systematic screening ensured that all eligible ICU patients were considered for inclusion. Written consent was obtained from all patients or their legal guardians. This study was approved by the Ethics Committee of the Medical Faculty of the Ruhr-University of Bochum (Registration no. 19-6606 3-BR). All research involving human participants was conducted in full accordance with the Declaration of Helsinki, as well as institutional and national guidelines and regulations. The study strictly adhered to the protocols outlined in the approved ethics vote. Intensive care patients aged 18 and older were eligible for recruitment if they met the current Sepsis-3 criteria for sepsis diagnosis (1). Individuals admitted with suspected or confirmed infection who did not initially meet the criteria for sepsis were not enrolled until sepsis-associated organ dysfunction developed. To enhance the generalizability of our findings and account for the heterogeneity of sepsis progression, the study protocol allowed for patient inclusion within 36 hours of sepsis diagnosis. This ensured that patients initially treated on the general ward before ICU transfer were not systematically excluded. For patients diagnosed with sepsis upon ICU admission, enrollment and sample collection were performed immediately to capture the earliest possible disease stage. Treatment of patients was carried out according to the current national and international guidelines and was not influenced by participation in the study. Blood samples for DNA, RNA, and serum analysis were collected within the first 36 hours after diagnosis and stored at -80 °C after initial processing. Blood samples for FACS analysis where freshly prepared and directly analyzed. A total of 278 patients with sepsis were successfully genotyped and were included in the genetic analyses. 30-day survival data were available for all 252 patients. Complete baseline clinical and demographic characteristics were available for 234 patients and are presented in **Table 1**. Flow cytometric (FACS) analyses were performed in a subset of 145 patients for whom data from freshly prepared blood samples were available. For gene expression analyses, RNA of sufficient quantity and quality was available from 131 patients. A detailed overview of patient inclusion and sample availability for each downstream analysis is provided in **Supplementary Figure 1**.

**Table 1:** Cohort description, classification according to rs8878 SNP AA and GG/AG-genotype.

Characteristics	AA (n= 46)	GG/AG (n=188)	P-value
Base characteristics			
Female sex, n (%)	18 (40.0)	75 (40.1)	1
Age, years (IQR)	64 (21.0)	64 (21.25)	0.642
SAPS-II, day 1 (IQR)	43 (33.5-52.5)	39 (28-49)	0.225
SOFA Score, day 1 (IQR)	6.00 (4.00-9.00)	8.00 (5.00-11.00)	0.393
Length of ICU stay, days (IQR)	16 (4-28)	9 (2-16)	**0.034**
Body temperature day 1 (°C)	31.002 ± 0.549	37.096 ± 0.906	0.305
Heart rate (beats per minute)	79 ± 17	84 ± 19	0.060
systolic blood pressure (SBP) (mmHg)	121 ± 12	122 ± 15	0.430
diastolic blood pressure (DBP) (mmHg)	62 ± 9	61 ± 9	0.333
mean arterial pressure (MAP) (mmHg)	83 ± 9	82 ± 10	0.197
Comorbid conditions, n (%)			
Hypertension	28 (60.9)	118 (62.8)	0.946
Cardiovascular disease	13 (28.3)	67 (35.6)	0.440
COPD*	3 (6.5)	23 (12.2)	0.431
Other Lung disease	8 (17.4)	22 (11.7)	0.430
Diabetes mellitus	13 (28.3)	59 (31.4)	0.816
Chronic kidney disease	6 (13)	41 (21.8)	0.261
Obesity (Body mass index ≥30 kg/m²)	20 (43.5)	56 (29.8)	0.109
Organ transplantation	2 (4.3)	24 (12.8)	0.122
Malignant neoplasms	9 (19.6)	41 (21.8)	0.895
Infection focus, n (%)			0.770
pulmonary	11 (24.4)	57 (30.3)	
Urinary tract	3 (6.7)	11 (5.9)	
Abdomen	5 (11.1)	29 (15.4)	
Central nervous system	1 (2.2)	3 (1.6)	
Bloodstream	1 (2.2)	6 (3.2)	
COVID-19	20 (44.4)	70 (37.2)	
Other/Unknown	4 (8.9)	12 (6.4)	
Laboratory values, day 1			
C-reactive protein [mg/L]	18.88 ± 14.10	15.49 ± 10.45	0.399
Procalcitonin [ng/mL]	14.06 ± 25.33	6.90 ± 13.53	0.550
Leucocytes [1000/µL]	16.33 ± 6.71	13.42 ± 7.23	0.213
Creatinine [mg/dL]	1.61 ± 1.06	1.53 ± 1.24	0.271
Bilirubin [mg/dL]	0.81 ± 0.98	0.93 ± 1.24	0.736
INF-α2	2.95 ± 2.5	2.28 ± 2.6	0.113
INF-γ	9.17 ± 9.17	6.59 ± 8.15	0.116

Data are presented as n (%) and median (IQR). *Chronic obstructive pulmonary disease (COPD).

Bold values show significance levels p < 0.05.

### 2.2 DNA genotyping

DNA was isolated from EDTA-blood samples using the my-Budget Blood DNA Midi Kit (Bio-Budget Technologies GmbH, Krefeld, Germany) according to the manufacturer’s instructions as previously described. Genotyping of the CXCL10 rs8878 was performed using the Thermo Fisher Scientific TaqMan^®^ SNP Genotyping Assay (Thermo Fisher Scientific, Wilmington, USA) and Bio-Rad CFX Connect Cycler Systems (Bio-Rad Laboratories, Inc., Hercules, USA) using a protocol of 95 °C for 10 minutes and 40 cycles of 95 °C for 15 seconds followed by 60 °C for 60 seconds.

### 2.3 RNA analysis

RNA was extracted from whole blood collected with Tempus™ Blood RNA Tubes (Applied Biosystems, Waltham, USA) using Tempus™ Spin RNA Isolation Reagent Kits (Applied Biosystems, Waltham, USA), followed by complementary DNA (cDNA) synthesis using the High-Capacity cDNA Reverse Transcription Kit by Applied Biosystems (Applied Biosystems, Waltham, USA). RNA concentration and purity were assessed using a NanoDrop spectrophotometer (Thermo Fisher Scientific, Wilmington, DE, USA). RNA samples were quantified spectrophotometrically, and purity was evaluated using the A260/A280 and A260/A230 absorbance ratios. Only RNA samples of sufficient quantity and quality were included in the subsequent gene expression analyses. Then, quantitative polymerase chain reaction was performed using our primers listed in **Supplementary File 2** for expression analysis of total CXCL10 in relation to ACTB, as previously described (13). The protocol used with GoTaq^®^ qPCR MasterMix (Promega, Madison, USA) involved 2 minutes of 95 °C followed by 40 cycles of 95 °C for 15 seconds and 60 °C for 60 seconds.

### 2.4 CXCL10 serum concentrations

Serum samples collected in Serum Gel Z tubes (Sarstedt, Germany) were analyzed using enzyme-linked immunosorbent assay (ELISA) kits to determine CXCL10 serum levels (Human CXCL10/IP-10 Quantikine ELISA Kit, bio-techne; Wiesbaden, Germany). Samples were diluted as necessary to fall within the standard detection range of the kit.

### 2.5 Plasma proteomics

Plasma samples were processed and analyzed as described previously by Palmowski et al. (14). Briefly, the samples were measured distributed over several batches and each batch was analyzed separately using DIA-NN (ver. 1.8.1), searching against the human SwissProt database (ver. 2022_05). Subsequently, a batch normalization procedure accompanied by quality control was carried out, resulting in log2-transformed normalized protein intensities. The resulting data set can be accessed via the PRIDE repository under the data set identifier PXD055932.

The respective cohort included 252 patients of which 30 carried the AA genotype. Propensity score matching was performed to select 60 control patients (1:2 ratio) matched according to sex and SOFA score. For each AA genotype patient two matched patients with the GG/AG-genotype were selected from the cohort, resulting in a comparison balanced for the considered confounders. Statistical differences in protein intensities between the two groups were assessed using student’s t-test and the resulting p-values were adjusted for multiple testing using the Benjamini-Hochberg method. Missing data was not imputed, only proteins with at least five observations in each of the compared sub-cohorts were considered for statistical testing. The effect size was calculated on back-transformed data as ratio of mean intensities. Calculations were performed in R (v.4.4.3) using the MatchIt package (15).

### 2.6 FACS analysis/immunophenotyping

EDTA-treated whole blood samples were stained with optimal concentrations of each antibody for 10 minutes at room temperature in the dark. Erythrocytes were lysed using VersaLyse (Beckman Coulter, USA) supplemented with 2.5% IOTest 3 Fixative Solution (Beckman Coulter, USA) for 30 minutes at room temperature in the dark. All samples were immediately acquired on a CytoFlex flow cytometer (Beckman Coulter, USA). Instrument performance was verified daily using CytoFlex Daily QC Fluorospheres (Beckman Coulter, USA) according to the manufacturer’s instructions. No adjustments to the compensation matrices were required throughout the study.

#### 2.6.1 Antibodies

The following fluorochrome-conjugated monoclonal antibodies were used for flow cytometric analysis. All antibodies were obtained from BioLegend (San Diego, CA, USA) unless otherwise indicated. CD4 (clone OKT4, A700, RRID: AB_571943, 1:200), CD3 (clone OKT3, BV785, RRID: AB_2563507, 1:200), CD8 (clone SK1, PeCy7, RRID: AB_2044006, 1:100).

### 2.7 Statistical analysis

Continuous variables are presented as means ± standard deviation (SD), or standard error of the mean (SEM) when normally distributed and as medians with interquartile ranges (IQR; 25th to 75th percentile) for distributions that are not normal. Differences between groups for continuous data were determined using the t-test, Mann-Whitney U testor the Wilcoxon rank-sum test, depending on the distribution of the data. Resulting p-values were further adjusted for multiple testing using the Benjamini-Hochberg false discovery rate (FDR). The 52 p-values were ranked in ascending order, and adjusted p-values were calculated according to the Benjamini–Hochberg method using Microsoft Excel or for proteomics in R (v.4.4.3) using the MatchIt package (15). For categorical variables, differences between groups were evaluated using either the Chi-square test or Fisher’s exact test, as appropriate. The distribution of genotypes was tested for Hardy-Weinberg equilibrium to ensure that genetic variation was consistent with expected frequencies using a chi-squared test. The primary survival endpoint was 30-day all-cause mortality. Survival time was defined as the interval between study inclusion and death from any cause within 30 days. Patients who survived beyond 30 days were censored at day 30.

Survival curves were constructed using the Kaplan-Meier analysis to estimate the time-dependent probability of 30-day survival. The log-rank test was applied to statistically compare these curves between the different groups and to identify significant differences in survival time. To evaluate the prognostic value of circulating T-cell counts, receiver operating characteristic (ROC) analysis and Youden index optimization were performed in an independent training cohort comprising 50 septic patients. Optimal cutoff values were determined separately for CD8^+^ and CD4^+^ T-cell counts. The resulting cutoffs were subsequently transferred without modification to an independent validation cohort of 93 septic patients, in which their association with 30-day survival was assessed using Kaplan-Meier analysis, log-rank testing, and multivariable Cox regression.

For the multivariable regression analysis, candidate variables were selected *a priori* based on biological plausibility, the study hypothesis, and established relevance in sepsis. Given the limited number of death events, the number of covariates was restricted to avoid overfitting. Collinearity among candidate variables was assessed, and only the strongest predictor among highly correlated variables was retained in the final model. To adhere to standard guidelines, we included a maximum of six variables for our 234-patient cohort (16). Multivariate Cox regression analysis was used to evaluate the influence of various predictors on survival, adjusting for potential confounders and quantifying hazard ratios. All statistical analyses were conducted using SPSS (version 28.0) and GraphPad Prism 8 or R.

## 3 Results

### 3.1 Baseline characteristics

A total of 234 patients with sepsis were included, comprising 46 individuals with the CXCL10 rs8878 AA-genotype and 188 with the GG/AG-genotype (**Table 1**). Baseline demographic and clinical parameters, including sex distribution, age, SAPS-II-, SOFA score, heart frequency, blood pressure and body temperature on day 1, as well as laboratory parameters such as cytokine concentrations did not differ significantly between genotype groups. However, patients with the AA-genotype exhibited a significantly longer ICU stay compared to carriers of the GG/AG-genotype (median 16 vs. 9 days, p = 0.034).

### 3.2 Association between CXCL10 rs8878 genotype and T cells

**Figure 1:**
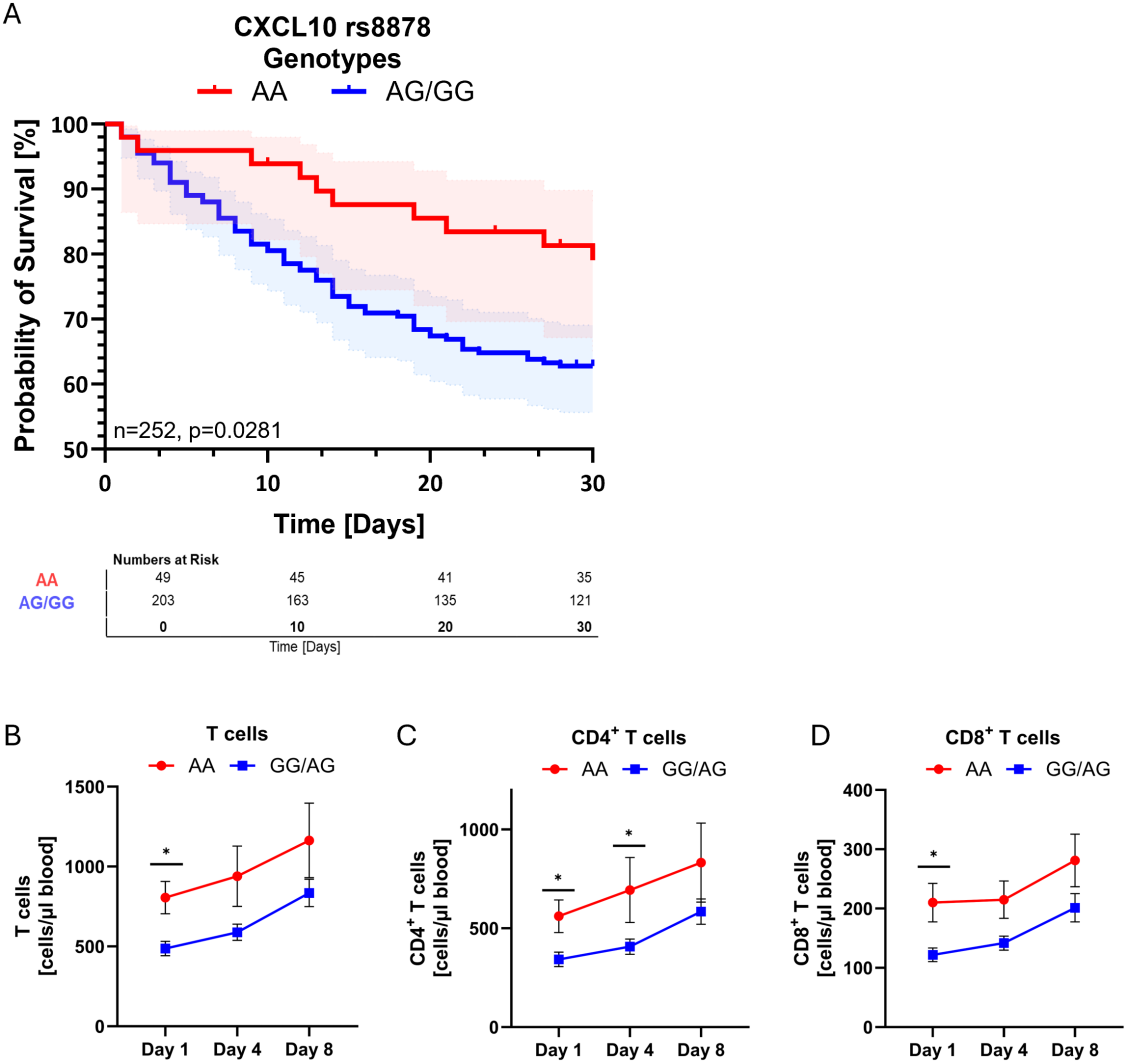
Impact of CXCL10 rs8878 genotype on circulating T cell counts on day 1. **(a)** Survival of septic patients stratified by CXCL10 rs8878 genotype. Kaplan-Meier curves show improved survival in A-allele carriers (AA) compared to AG/GG carriers (log-rank p = 0.0281). The proportion of survivors was 79% in AA carriers versus 62% in AG/GG carriers. **(b)** Total T cell counts **(b)**, CD4+ T cell counts **(c)** and CD8+ T cells count **(d)** were significantly higher in AA genotype carriers compared to AG/GG carriers. Data are presented as mean ± SEM. Statistical significance was assessed using the Mann-Whitney U test. Sample sizes were n = 145 (day 1), n = 119 (day 4), and n = 83 (day 8).). *p < 0.05.

Next, we examined the impact of the CXCL10 rs8878 genotype on T cell counts. Patients carrying the A allele (AA) showed significantly higher T lymphocyte numbers compared to AG and GG genotypes (mean 805.5 cells/µL vs 486.9 cells/µL; p = 0.0202) on day 1 (**Figure 1a**). A similar effect was observed for CD4^+^ T cells as well as for CD8^+^ T cells, with AA genotype carriers exhibiting higher counts than AG/GG carriers (CD4^+^ T cells: mean 561.2 cells/µL vs 342.9 cells/µL; p = 0.0113; **Figure 1c**; CD8^+^ T cells: mean 210.1 cells/µL vs 122.0 cells/µL; p = 0.0452; **Figure 1d**). In line with this, significant differences between genotypes were also detected across multiple T cell subsets, including CD4^+^ and CD8^+^ T cell compartments and their respective naïve, central memory, effector memory, and TEMRA subsets, as well as NKT cells, on day 1 (**Table 2**). In contrast, no significant differences were observed for CD3^+^CD56^+^ cells, B cells, NK cells (including CD56^bright^ and CD56^dim^ subsets), total leukocytes, granulocytes, or monocyte populations, including CD14^+^ monocytes as well as classical, intermediate, and non-classical monocytes.

**Table 2:** Distribution of circulating immune cell counts stratified by CXCL10 rs8878 genotype on day 1 of sepsis.

Cell type	AA Cell count per µl blood	GG/AG Cell count per µl blood	Adjusted p-value
Lymphocytes day 1	1168.49	646.19	0.0114
NKT cells day 1	34.59	12.59	0.0387
T cells day 1	637.45	359.79	0.0140
CD4 T cells day 1	460.96	280.35	0.0307
CD4 CM cells day 1	118.54	65.05	0.0277
CD4 EM cells day 1	106.10	57.84	0.0325
CD4 Naive cells day 1	156.48	89.60	0.0338
CD4 CD8 T cells day 1	16.86	11.64	0.0462
CD8 T cells day 1	154.18	82.19	0.0215
CD8 CM cells day 1	32.22	19.02	0.0271
CD8 EM cells day 1	27.46	13.53	0.0280
CD8 Naive cells day 1	61.19	27.22	0.0264
CD8 TEMRA cells day 1	42.75	16.84	0.0250

P-value were adjusted according to Benjamini Hochberg. Significant differences between the genotypes are displayed. Data are presented as median cell count per µl blood.

### 3.3 CXCL10 rs8878 genotype and 30-day survival

To determine the clinical relevance of the rs8878 genotype, we performed 30-day survival analyses. Kaplan-Meier curves demonstrated improved 30-day survival among A-allele carriers (AA) compared to AG/GG carriers (log-rank p = 0.0281, **Figure 1c**). The proportion of survivors was 79% in AA carriers versus 62% in AG/GG carriers. This 30-day survival advantage is consistent with the observed higher T cell counts in AA carriers. Notably, no associations were observed between genotype and routine vital parameters. Given the observed association between CXCL10 rs8878 genotype and both T cell counts and 30-day survival, we next investigated whether circulating T cell levels themselves are directly associated with patient outcomes.

### 3.4 T cell counts and 30-day survival

**Figure 2:**
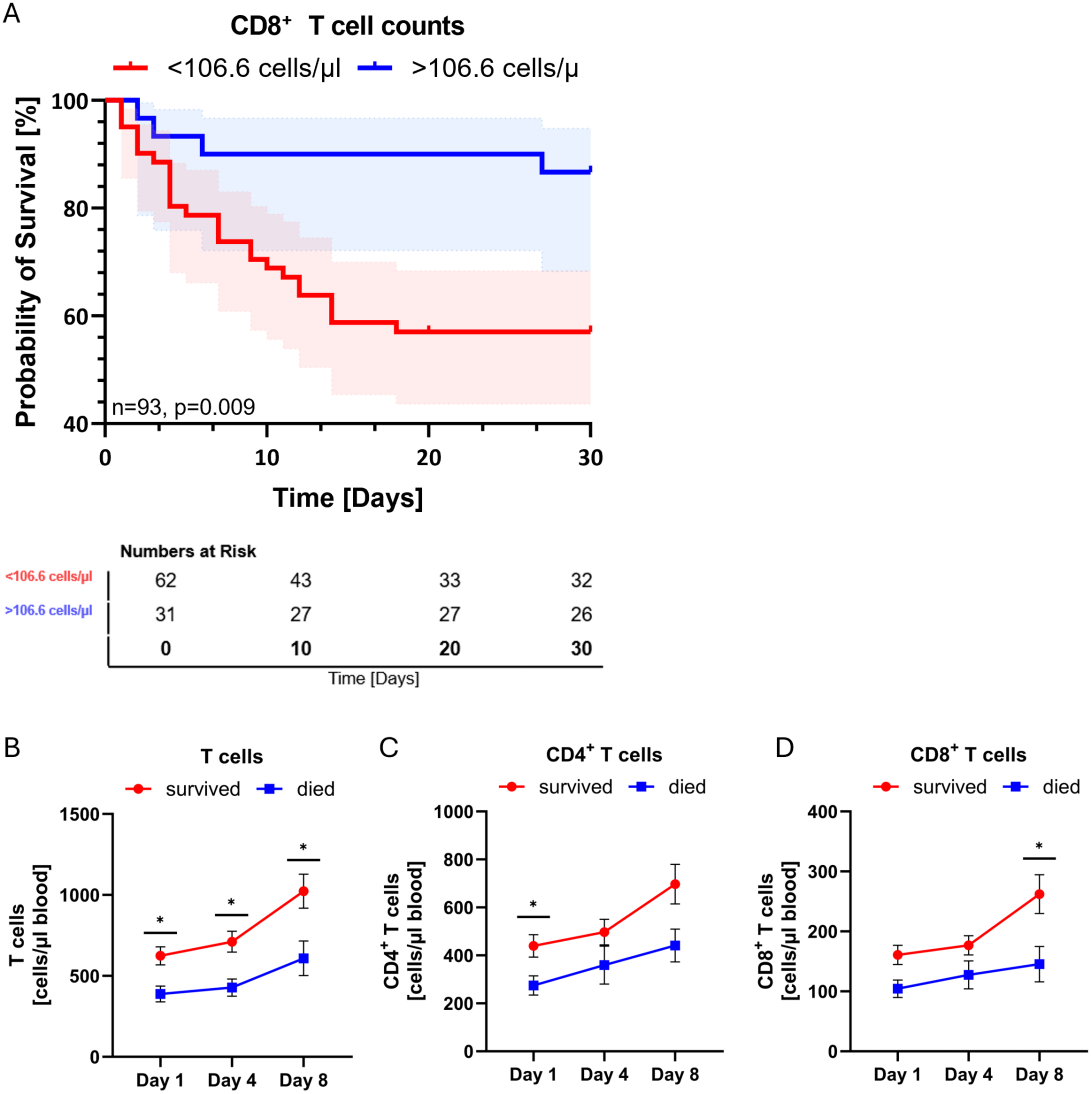
Circulating T cell counts and survival in septic patients. **(a)** Kaplan-Meier survival analysis of the validation cohort stratified according to the CD8^+^ T-cell cutoff identified by ROC analysis. Patients with CD8^+^ T-cell counts >106 cells/µL exhibited significantly improved 30-day survival compared with patients below the cutoff (log-rank p = 0.009, n=93). **(b)** Total T-cell counts were significantly higher in survivors compared with non-survivors on days 1, 4, and 8. **(c)** CD4^+^ T-cell counts were significantly higher in survivors on day 1 and showed a trend towards higher values on day 8 (p=0.0577, whereas no significant difference was observed on day 4. **(d)** CD8^+^ T-cell counts were consistently higher in survivors than in non-survivors throughout the observation period and reached statistical significance on day 8. Data in panels **(B–D)** are presented as mean ± SEM. Statistical significance was assessed using the Mann-Whitney U test. Sample sizes were n = 145 (day 1), n = 119 (day 4), and n = 83 (day 8). *p < 0.05.

Circulating T cell counts were significantly higher in survivors compared to non-survivors of sepsis. As shown in **Figure 2**, survivors exhibited a mean T cell count of 623.0 cells/µL versus 388.2 cells/µL in non-survivors (p = 0.0051) on day 1, survivors exhibited a mean T cell count of 711.0 cells/µL versus 427.6 cells/µL in non-survivors (p = 0.0029) on day 4 and survivors exhibited a mean T cell count of 1024 cells/µL versus 608.7 cells/µL in non-survivors (p = 0.0226) on day 8 (**Figure 2b**).

Differences between survivors and non-survivors were also observed for circulating T-cell subsets. For CD4^+^ T cells, survivors exhibited significantly higher cell counts on day 1 compared with non-survivors (mean difference: 164.7 cells/µL, 95% CI 16.8–312.6, p = 0.0238). No significant differences were observed on day 4 (mean difference: 137.5 cells/µL, 95% CI −96.6 to 371.7, p = 0.3953), whereas a trend towards higher CD4^+^ T-cell counts in survivors was observed on day 8 (mean difference: 255.8 cells/µL, 95% CI −6.2 to 517.7, p = 0.0577; **Figure 2c**).

A similar pattern was observed for CD8^+^ T cells. Survivors exhibited higher CD8^+^ T-cell counts than non-survivors on day 1 (160.8 vs. 104.4 cells/µL, p = 0.1719) and day 4 (176.0 vs. 125.9 cells/µL, p = 0.377), although these differences did not reach statistical significance. By day 8, CD8^+^ T-cell counts were significantly higher in survivors than in non-survivors (249.4 vs. 140.3 cells/µL, p = 0.0195; **Figure 2d**).

To determine whether a clinically relevant cutoff could distinguish surviving from non-surviving septic patients, receiver operating characteristic (ROC) analysis and Youden index optimization were initially performed in a training cohort comprising 50 septic patients using CD8^+^ T-cell counts. This analysis identified an optimal cutoff of 106.6 CD8^+^ T cells/µl blood, corresponding to a sensitivity of 72.7% and a specificity of 80.6%. The cutoff was subsequently transferred to an independent validation cohort of 93 septic patients.

In the validation cohort, patients with CD8^+^ T-cell counts above the cutoff exhibited significantly improved 30-day survival compared with patients below the cutoff (83.9% vs. 56.5%; log-rank p < 0.001; **Figure 2A**). In multivariable Cox regression analysis adjusted for SOFA score, age, and sex, a CD8^+^ T-cell count ≤106.6 cells/µl remained an independent predictor of mortality, corresponding to a 3.8-fold increased risk of death (hazard ratio 3.83, 95% CI 1.41–10.42, p = 0.009; **Table 3**).

**Table 3:** Multivariate COX-regression of CD8^+^-cell count cutoff.

Variable	p-value	Hazard ratio	95,0% confidence interval	
			lower	upper
SOFA score	<0.001	1.397	1.236	1.580
Age	0.715	1.005	0.979	1.032
Sex	0.323	1.510	0.667	3.420
CD8 T-cell count ≤106.6 cells/µl	0.009	3.83	1.41	10.42

The same approach was applied to CD4^+^ T-cell counts. However, no significant association between the corresponding CD4^+^ T-cell cutoff and 30-day survival was observed in the validation cohort (log-rank p = 0.363).

### 3.5 CXCL10 mRNA expression and protein serum concentration in non-survivors

**Figure 3:**
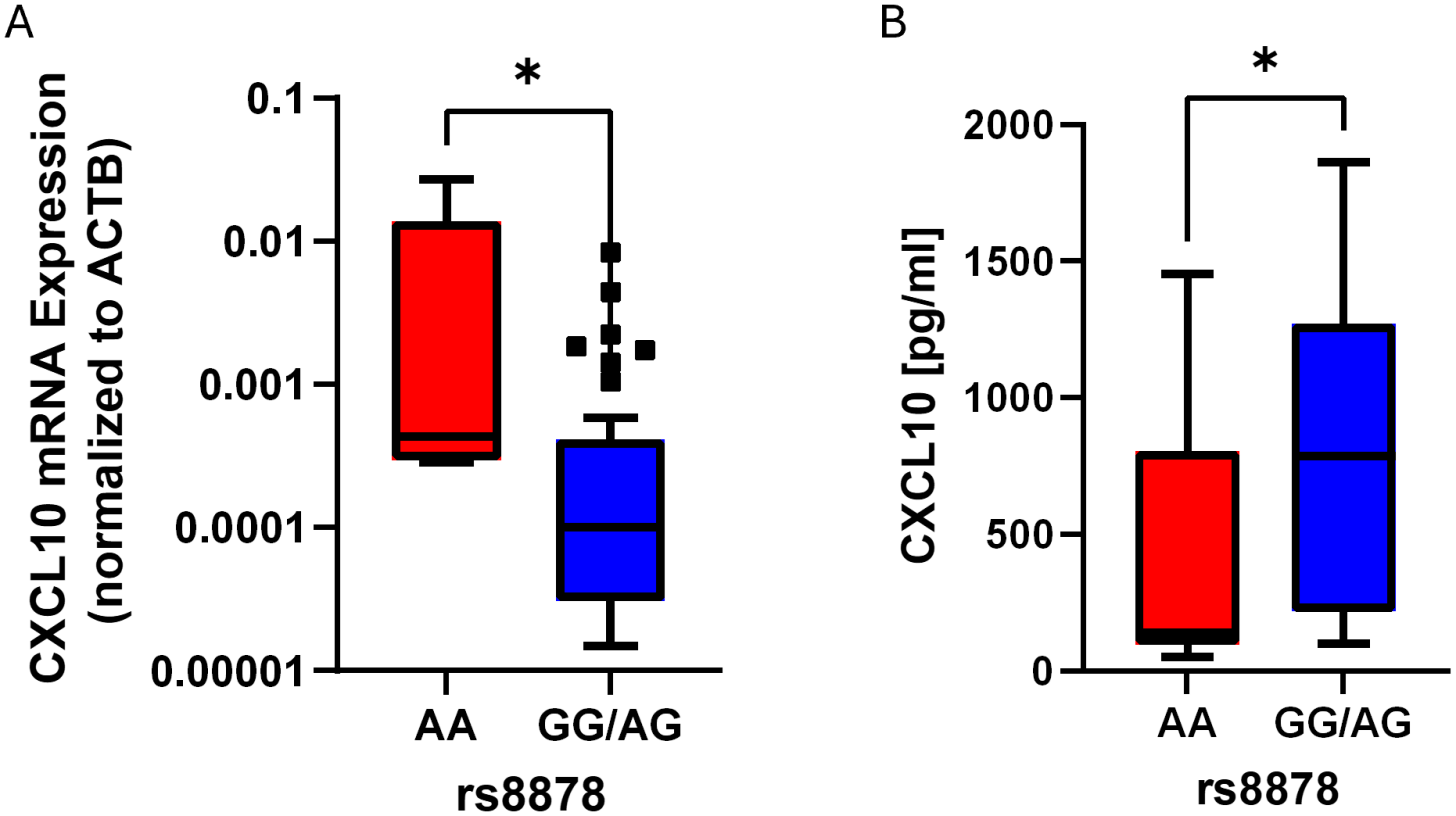
CXCL10 expression according to rs8878 genotype in non-surviving septic patients. **(a)** CXCL10 mRNA levels were significantly higher in AA-genotype carriers (n=5) compared to AG/GG carriers (n=37) (median 0.00043 vs 0.00010 relative units; p = 0.0259, Mann-Whitney U test). **(b)** In contrast, circulating CXCL10 protein concentrations were lower in AA-carriers (n=5) than in G-allele carriers (n=27) (median 438 139.5 pg/mL vs 785.6 pg/mL; p = 0.0349, Mann-Whitney U test). *p < 0.05.

Finally, we assessed CXCL10 mRNA expression in all patients. Interestingly, a difference was only observed in non-surviving patients. AA-genotypes exhibited significantly higher CXCL10 transcript levels compared to AG/GG-genotypes (median 0.00043 vs 0.00010 relative units; p = 0.0259; **Figure 3a**). An opposite effect was detected in CXCL10 serum concentration: G-allele carriers showed a median concentration of 785.6 pg/ml and AA-genotype 139.5 pg/ml (p=0.0349; **Figure 3b**).

### 3.6 Correlation analysis of cytokines with CXCL10 concentration

**Figure 4:**
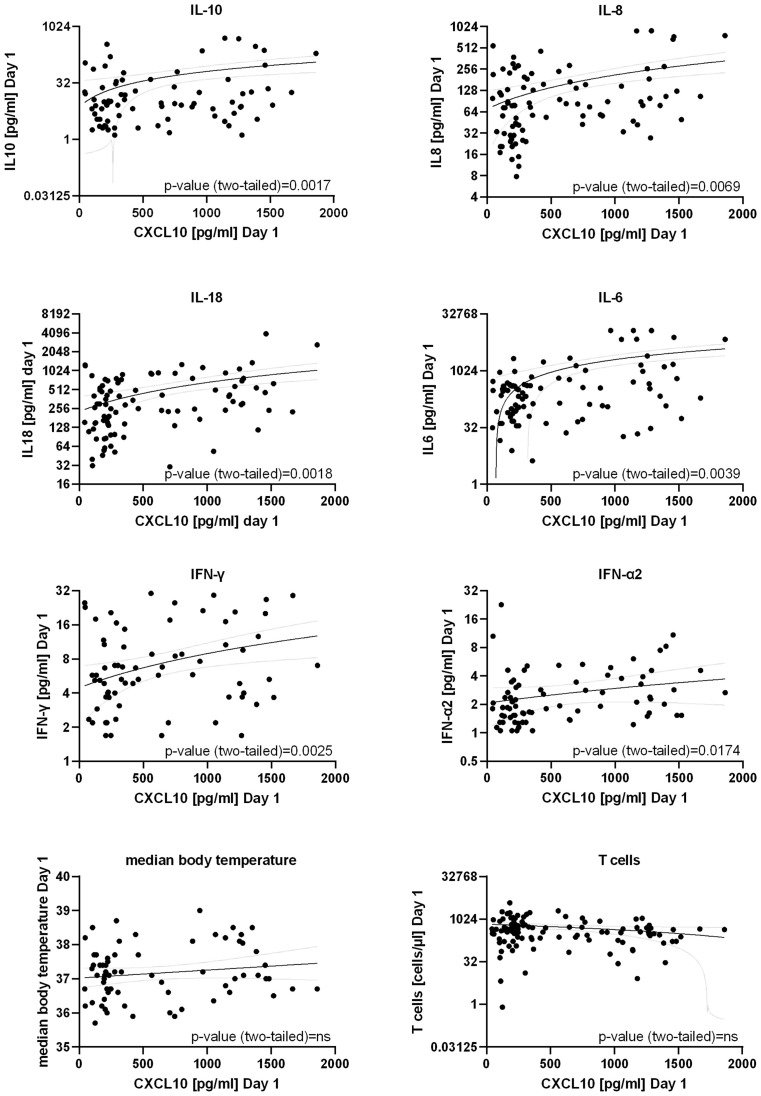
Scatter plots illustrating the relationships between CXCL10 concentrations and circulating cytokine levels, body temperature, and total T cell counts. Spearman correlation analysis revealed that CXCL10 concentrations on day 1 were positively correlated with IL-10 (r = 0.3268, p = 0.0017, n=90), IL-8 443 (r = 0.2847, p = 0.0069, n=89), IL-18 (r = 0.3232, p = 0.0018, n=91), IL-6 (r = 0.3001, p = 0.0039, n=91), IFN-γ (r = 444 0.3133, p = 0.0025, n=91), and interferon-α2 (r = 0.2488, p = 0.0174, n=91). No significant correlation was observed 445 with body temperature (r = 0.1539, p = 0.2102, n=68), whereas CXCL10 concentrations were inversely correlated with total T cell counts (r = -0.2159, p = 0.0235, n=110).

Correlation analyses were performed to assess the relationship between CXCL10 concentrations and circulating cytokine levels. On day 1, CXCL10 concentrations showed significant positive correlations with IL-10 (r = 0.3268, p = 0.0017), IL-8 (r = 0.2847, p = 0.0069), IL-18 (r = 0.3232, p = 0.0018), IL-6 (r = 0.3001, p = 0.0039), MCP-1 (r= 0.2840; p= 0,0067), IFN-γ (r = 0.3133, p = 0.0025), and interferon-α2 (r = 0.2488, p = 0.0174; **Figure 4**). No significant correlation was observed between CXCL10 levels and body temperature (r = 0.1539, p = 0.2102). In contrast, CXCL10 serum concentrations were inversely correlated with total T cell counts (r = -0.2159, p = 0.0235; **Figure 4**).

### 3.7 Plasma proteome is not associated with rs8878 genotypes

The plasma protein composition of the patients’ groups with AA-genotype and GG/AG-genotype at day 1 was assessed. After identifying 429 protein groups we did not observe any significant changes between the two groups (see **Supplementary Table 1**).

### 3.8 Summary of findings

In this study, we demonstrate that the CXCL10 rs8878 genotype is associated with both immune cell composition and clinical outcome in sepsis. Circulating T cell counts were significantly higher in survivors compared to non-survivors across multiple time points, indicating a robust association between preserved T cell levels and improved outcome. This effect was particularly evident for CD8^+^ T cells, for which a cutoff value of 106 cells/µL was identified, above which patients showed significantly improved 30-day survival. Importantly, this cutoff remained an independent predictor of survival in multivariate analysis.

In line with these findings, carriers of the AA genotype exhibited higher circulating T cell counts, particularly within the CD8^+^ compartment, and showed significantly improved 30-day survival compared to G-allele carriers. These genotype-dependent differences were consistently observed across multiple T cell subsets, while innate immune cell populations and routine vital parameters remained unaffected. Furthermore, CXCL10 expression analyses revealed a distinct pattern in non-survivors, with increased mRNA levels but reduced circulating protein concentrations in AA genotype carriers, suggesting complex regulatory mechanisms. In addition, CXCL10 concentrations on day 1 were positively associated with multiple pro- and anti-inflammatory cytokines, showed no relationship with clinical routine vital parameters, and were inversely correlated with total T cell counts.

Together, these findings support a role for CXCL10 rs8878 in modulating adaptive immunity, particularly T cell responses, and influencing survival in sepsis.

## 4 Discussion

Our data indicate that the CXCL10 rs8878 genotype defines a distinct immunological phenotype of sepsis. Patients carrying the AA genotype were characterized by better preserved T cell responses and a more favorable clinical outcome. In contrast, the G-allele-associated phenotype was marked by pronounced T cell loss and significantly higher mortality.

In this study, we also demonstrate that circulating T cell counts are a strong predictor of 30-day survival in septic patients, underscoring the critical role of adaptive immunity in determining sepsis outcomes. Patients with higher T cell numbers exhibited significantly improved survival, in line with previous reports identifying lymphopenia as a hallmark of sepsis-associated immune dysfunction (17).

These findings suggest that genetic variation in CXCL10 contributes to heterogeneity in the immune response during sepsis and is associated with differences in adaptive immunity and clinical outcome (4, 18).

To the best of our knowledge, this is the first study to directly link a specific genetic polymorphism with both alterations in T cell counts and clinical outcomes in sepsis.

Previous studies investigating genetic determinants of sepsis predominantly focused on associations between single nucleotide polymorphisms and clinical endpoints such as mortality, organ dysfunction, or secondary infections, without incorporating direct immunological phenotyping of T cell quantity or function. Our findings therefore provide novel evidence connecting genetic variation, adaptive immune dysregulation, and sepsis outcome within a single integrated analysis. In particular, variants in immune checkpoint genes such as *TIM-3* (19), *CTLA-4* (20), and *PD-1* (21) have been linked to divergent effects on mortality, likely reflecting their regulatory roles in T cell activation and apoptosis. Similarly, polymorphisms in pattern recognition receptors (e.g., *TLR1* (22), *TLR4*) and inflammatory mediators (e.g., *TNF-α*) have been associated with increased susceptibility to and severity of sepsis (23).

Importantly, we identified a genotype-dependent effect of CXCL10 rs8878 on T cell abundance. Carriers of the AA-genotype displayed higher T cell counts and enhanced 30-day survival compared to AG/GG-genotypes. These findings suggest that genetic variation at CXCL10 may modulate immune competence in septic patients, potentially by influencing T cell trafficking or proliferation. This supports the concept that host genetic factors contribute to the heterogeneity of immune responses in sepsis (24–26).

In line with this, we observed a broad and simultaneous reduction of multiple lymphocyte populations in carriers of the AG/GG genotypes already at day 1 of sepsis. Specifically, decreased cell numbers were evident across innate-like and adaptive compartments, including NKT cells, total T cells, as well as all major CD4^+^ and CD8^+^ T cell subsets. This encompassed central memory, effector memory, naïve, and TEMRA populations, indicating a global disturbance of T cell homeostasis rather than a subset-specific effect.

Such a pan-lymphocyte depletion reflects a profound state of systemic immune failure (27, 28). Mechanistically, this phenomenon is likely driven by converging processes, including apoptosis, cytokine storm-induced cell death, redistribution and sequestration of lymphocytes into peripheral tissues, and active immunosuppressive signaling pathways (29). Beyond the quantitative loss, qualitative alterations in T cell composition are also highly relevant, with a shift toward exhausted and regulatory phenotypes characterized by increased expression of inhibitory receptors and reduced effector functionality (30).

Interestingly, despite the observed genotype-dependent differences in T cell abundance, we did not detect a significant effect of the CXCL10 rs8878 polymorphism on cytokine levels in our cohort. This suggests that the impact of this SNP may be more specifically related to cellular immune dynamics rather than systemic cytokine responses. One possible explanation is that CXCL10 variation primarily influences leukocyte trafficking, tissue homing, or cell survival, rather than directly modulating circulating cytokine concentrations. Alternatively, cytokine measurements in plasma may not adequately reflect localized immune processes or compartment-specific effects, particularly within tissues where T cell redistribution occurs. Notably, CXCL10 concentrations were positively correlated with multiple pro- and anti-inflammatory cytokines and inversely associated with total T cell counts, indicating that CXCL10 is closely linked to systemic inflammatory activity and T cell depletion. Together, these findings support a model in which genetic variation in CXCL10 does not directly alter circulating cytokine levels but may modulate the cellular response to an inflammatory milieu, thereby influencing T cell homeostasis and clinical outcome.

The clinical implications of this widespread lymphocyte reduction are considerable. Early depletion of T cell populations has been consistently associated with impaired host defense, increased susceptibility to secondary infections, and adverse outcomes in sepsis (31). In this context, our findings suggest that the AG/GG genotypes may predispose patients to an exaggerated and early immunosuppressive phenotype, thereby contributing to poorer clinical trajectories.

Taken together, the observed genotype-dependent differences in T cell abundance, combined with the extensive depletion across all major T cell subsets, point toward a role of CXCL10 genetic variation in shaping both the magnitude and quality of the adaptive immune response in sepsis. This highlights a potential mechanistic link between host genetics, early immune dysregulation, and outcome, and underscores the importance of integrating immunophenotyping with genetic data to better understand sepsis heterogeneity (14, 32, 33).

Among non-survivors, AA-genotypes carriers exhibited higher CXCL10 mRNA expression, indicating a possible mechanistic link between rs8878 genotype and immune regulation. CXCL10 is known to recruit activated T cells to sites of infection and inflammation, and elevated expression may enhance effector T cell responses, which could contribute to improved survival in AA-genotype (7). Notably, CXCL10 concentrations were positively correlated with multiple pro- and anti-inflammatory cytokines, including IL-6, IL-8, IL-10, IL-18, MCP-1, IFN-γ, and interferon-α2, while showing an inverse correlation with total T cell counts. These associations suggest that CXCL10 acts as a key integrator of systemic inflammatory activity and adaptive immune cell dynamics. Our findings indicate that rs8878 may indirectly influence both T cell abundance and chemokine expression, linking genetic variation, inflammatory milieu, and immune cell homeostasis to clinical outcomes in sepsis.

Interestingly, the increased CXCL10 mRNA expression observed in AA-genotype carriers was not paralleled by higher circulating CXCL10 protein concentrations. In contrast, AA carriers exhibited lower serum CXCL10 levels despite increased transcript abundance. This apparent discordance suggests that the relationship between CXCL10 transcription and circulating protein levels is complex and may be influenced by post-transcriptional and post-translational regulatory mechanisms. Several explanations are conceivable, including differences in mRNA stability, translational efficiency, protein secretion, receptor-mediated consumption, or protein clearance. Furthermore, whole-blood mRNA expression and circulating serum protein concentrations reflect distinct biological compartments and may originate from different cellular sources. Similar discrepancies between transcript and protein abundance have been described for other inflammatory mediators and highlight the complexity of immune regulation during sepsis. Future mechanistic studies are required to determine how rs8878 influences CXCL10 regulation at the transcriptional and post-transcriptional level.

About the precise role of the SNP in CXCL10 function, we can only speculate. Some studies examining this polymorphism already exist. The rs8878 SNP in the CXCL10 gene shows distinct associations across disease states. In type 1 diabetes, homozygosity for the A-allele associates with a protective effect (odds ratio 0.48, 95% confidence interval 0.23-0.99) (34, 35). In rheumatoid arthritis, the G-allele confers an increased risk (odds ratio 1.49, 95% confidence interval 1.03-2.13, p = 0.034), with the GG-genotype also linked to a higher likelihood of developing extra-articular manifestations (odds ratio 1.75, 95% confidence interval 1.04-3.03) (12). In colorectal cancer, the A-allele appears more frequently in patients (52.6% versus 47.6% in controls) and associates with increased risk (odds ratio 1.22, 95% confidence interval 1.01-1.47, p = 0.037), along with elevated CXCL10 protein levels (36).

So far, little is known about the functional impact of the rs8878 variant. Based on its genomic location in the 3′-untranslated region (3′ UTR) of CXCL10 (exon 4), one can only surmise about its possible effects. Variants within the 3′ UTR may influence mRNA stability or microRNA binding, thereby modulating CXCL10 protein expression. In a study on colorectal cancer, rs8878 was discussed as a potential regulator through such post-transcriptional mechanisms (36).

Several single nucleotide polymorphisms (SNPs) in the CXCL10 gene have been explored. The -135G>A promoter variant (rs56061981) affects promoter activity through NF-κB transactivation and has been linked to protection against tuberculosis and increased susceptibility to hepatitis B infection (37) or malaria (11). Moreover, tag-SNPs including rs4508917, rs56061981, and rs56316945 have been associated with altered promoter function and variable disease risk in chronic obstructive pulmonary disease (38) and a 3′-UTR variant (rs3921) has been associated with survival following bone marrow transplantation (39).

This study has several implications. First, it highlights the potential of CXCL10 genotyping as a prognostic biomarker in sepsis, identifying patients with a more favorable immunologic profile. Second, the observed association between CXCL10 genotype and T cell abundance underscores the relevance of adaptive immunity in sepsis outcomes and may inform the development of immunomodulatory therapies. Finally, our results provide a framework for future studies investigating the functional consequences of CXCL10 genetic variation, including mechanistic studies on chemokine signaling and T cell trafficking.

Several limitations should be acknowledged. Our study cohort, while well-characterized, is relatively modest in size and we have not included a validation cohort, which may limit generalizability. CXCL10 mRNA expression was only assessed in whole blood samples, and protein-level analyses were only performed in serum. In addition, longitudinal measurements were not performed for protein and mRNA analyses. Additionally, while the association between rs8878, T cells, and survival is compelling, causality cannot be established in this observational study. A further limitation of this study is that the exact timing of blood sampling relative to sepsis diagnosis and initiation of antibiotic therapy was not considered in the analyses. Although all samples were obtained within 36 hours after sepsis diagnosis and patients received standard-of-care treatment according to current guidelines, temporal changes in immune responses during this period may have influenced the observed immunological and transcriptional profiles. Future studies should incorporate precise sampling time points and longitudinal analyses to better capture the dynamic immune response during sepsis. Another limitation of this study is that detailed ancestry information and genome-wide genetic data were not available, precluding formal assessment of population stratification. Although all patients were recruited from participating centers within Germany and are therefore expected to represent a relatively homogeneous Central European population, residual population substructure cannot be excluded and may have influenced the observed genotype–phenotype associations. Future studies should incorporate ancestry-informed analyses to account for potential population stratification. Another limitation of this study is the lack of a non-septic control cohort. Consequently, it cannot be determined whether the observed associations between CXCL10 rs8878 genotype, CXCL10 expression, and T-cell abundance are specific to the septic response or reflect baseline genotype-dependent differences that are also present in healthy individuals. Future studies including healthy controls and additional disease cohorts are warranted to clarify the context specificity of these findings. As in all longitudinal sepsis studies, analyses performed at later time points are potentially affected by survivor bias, as only patients who survived and remained available for follow-up sampling could contribute data. To minimize this effect, all survival analyses were based on day 1 measurements. Nevertheless, longitudinal comparisons at days 4 and 8 should be interpreted with appropriate caution.

In conclusion, the CXCL10 rs8878 AA-genotype is associated with higher circulating T cell counts and improved survival in sepsis, likely mediated through enhanced CXCL10 expression. These findings emphasize the interplay between host genetics and adaptive immunity in sepsis and support further investigation into CXCL10 as both a prognostic biomarker and a potential target for immunomodulatory interventions.

## Data Availability

The mass spectrometry proteomics data have been deposited to the ProteomeXchange Consortium via the PRIDE partner repository with the dataset identifier PXD064125. All other data are available from the corresponding author on reasonable request.
