# Supplementary material for: CXCL10 rs8878 identifies a genotype-associated immune phenotype linked to T-lymphocyte preservation and survival in sepsis
Source: Front Immunol. 2026 Jul 17;17:1887361. doi: 10.3389/fimmu.2026.1887361 (PMC13424974; doi:10.3389/fimmu.2026.1887361)
Supplement: Supplementary file 2 [file Table2.docx]

Supplementary Table 2: distribution of circulating immune cell counts stratified by CXCL10 rs8878 genotype on day 1 and day 4 of sepsis. Data are presented as median cell count per µl blood. P-value were adjusted according to Benjamini HochbergTable

| **Cell Type** | **AA**  **Cell count per µl blood** | **GG/AG**  **Cell count per µl blood** | **Adjusted p-Value** |
| --- | --- | --- | --- |
| Leucocytes day 1 | 12100.81 | 12088.38 | 0.9028 |
| Granulocytes day 1 | 8866.68 | 10235.64 | 0.5380 |
| *Lymphocytes day 1* | *1168.49* | *646.19* | **0.0114** |
| CD3CD56 day 1 | 184.16 | 146.14 | 0.1237 |
| B cells day 1 | 130.11 | 73.35 | 0.1980 |
| NK cells day 1 | 102.83 | 61.10 | 0.0675 |
| CD56 Bright cells day 1 | 4.80 | 3.30 | 0.1682 |
| CD56 Dim cells day 1 | 87.31 | 50.81 | 0.0883 |
| *NKT cells day 1* | *34.59* | *12.59* | **0.0387** |
| *T cells day 1* | *637.45* | *359.79* | **0.0140** |
| *CD4 T cells day 1* | *460.96* | *280.35* | **0.0307** |
| *CD4 CM cells day 1* | *118.54* | *65.05* | **0.0277** |
| *CD4 EM cells day 1* | *106.10* | *57.84* | **0.0325** |
| *CD4 Naive cells day 1* | *156.48* | *89.60* | **0.0338** |
| CD4 TEMRA cells day 1 | 13.57 | 7.85 | 0.0927 |
| *CD4 CD8 T cells day 1* | *16.86* | *11.64* | **0.0462** |
| *CD8 T cells day 1* | *154.18* | *82.19* | **0.0215** |
| *CD8 CM cells day 1* | *32.22* | *19.02* | **0.0271** |
| *CD8 EM cells day 1* | *27.46* | *13.53* | **0.0280** |
| *CD8 Naive cells day 1* | *61.19* | *27.22* | **0.0264** |
| *CD8 TEMRA cells day 1* | *42.75* | *16.84* | **0.0250** |
| CD14 Monocytes day 1 | 1813.23 | 1298.45 | 0.1808 |
| Classical Monocytes day 1 | 1583.81 | 1127.60 | 0.1788 |
| Intermediate Monocytes day 1 | 22.23 | 23.13 | 0.6713 |
| Nonclassica Monocytes cell day 1 | 4.28 | 5.90 | 0.7204 |
| Leucocytes cells day 4 | 11242.11 | 10887.64 | 0.8942 |
| Granulocytes cells day 4 | 8315.83 | 8233.81 | 0.8045 |
| *Lymphocytes cells day 4* | *1090.94* | *804.41* | **0.0402** |
| CD3 CD56 cells day 4 | 212.97 | 164.38 | 0.2086 |
| B cells day 4 | 126.46 | 96.55 | 0.1882 |
| NK cells day 4 | 90.18 | 78.78 | 0.0675 |
| CD56 Bright cells day 4 | 3.54 | 3.70 | 0.6287 |
| CD56 Dim cells day 4 | 84.93 | 71.19 | 0.1245 |
| NKT cells day 4 | 33.22 | 17.84 | 0.1217 |
| T cells day 4 | 720.68 | 527.48 | 0.0504 |
| CD4 T cells day 4 | 457.98 | 339.43 | 0.0654 |
| CD4 CM cells day 4 | 142.51 | 81.80 | 0.0786 |
| CD4 EM cells day 4 | 99.87 | 64.98 | 0.1206 |
| CD4 Naive cells day 4 | 202.31 | 121.04 | 0.0881 |
| CD4 TEMRA cells day 4 | 11.43 | 11.36 | 0.2606 |
| CD4 CD8 T cells day 4 | 18.63 | 12.75 | 0.0767 |
| CD8 T cells day 4 | 179.93 | 104.47 | 0.0672 |
| CD8 CM cells day 4 | 38.62 | 26.60 | 0.0665 |
| CD8 EM cells day 4 | 28.81 | 16.27 | 0.1206 |
| CD8 Naive cells day 4 | 68.01 | 37.18 | 0.1212 |
| CD8 TEMRA cells day 4 | 60.98 | 32.32 | 0.1069 |
| CD14 Monocytes day 4 | 2091.84 | 1582.21 | 0.2731 |
| Classical Monocytes day 4 | 1882.24 | 1402.90 | 0.2034 |
| Intermediate Monocytes day 4 | 16.00 | 15.36 | 0.9400 |
| Nonclassica Monocytes day 4 | 7.05 | 6.00 | 0.9155 |
